# Determinants of burnout syndrome among nursing students in Cameroon: cross-sectional study

**DOI:** 10.1186/s13104-018-3567-3

**Published:** 2018-07-09

**Authors:** Tsi Njim, Clarence Mbanga, Dave Mouemba, Haman Makebe, Louis Toukam, Belmond Kika, Isabelle Mulango

**Affiliations:** 1 Health and Human Development (2HD) Research Group, Douala, Littoral region Cameroon; 2 Mankon Sub-divisional Hospital, Bamenda, North west region Cameroon; 3 Regional Hospital Annex Kousseri, Garoua, Far north region Cameroon; 4 Faculty of Health Sciences, University of Buea, Buea, South west region Cameroon; 5 Faculty of Health Sciences, University of Bamenda, Bamenda, North west region Cameroon; 6 District Hospital Ekondo-Titi, Ekondo-Titi, South west region Cameroon; 7 District Hospital Kumba, Kumba, South west region Cameroon

**Keywords:** Burnout syndrome, Nursing students, Cameroon, OLdenburg Burnout Inventory

## Abstract

**Objectives:**

Burnout syndrome defined as a state of emotional exhaustion and disengagement; which could reduce optimal healthcare delivery, is relatively common amongst healthcare trainees. We sought to assess the determinants of burnout syndrome amongst nursing students in Cameroon. A cross-sectional study which included 447 nursing students recruited after written informed consent by convenience sampling, was carried out from January to April 2018. A printed self-administered questionnaire assessing burnout using the OLdenburg Burnout Inventory was used. Multivariable linear regression was used to identify independent determinants of burnout syndrome.

**Results:**

Most (81.17%) of the students were female with the average for disengagement items being 17.10 ± 3.09 (minimum = 8, maximum = 26) and 20.94 ± 3.04 (minimum = 13, maximum = 31) for exhaustion items. After multivariable linear regression analysis, satisfaction with results (RC: − 1.42, 95% CI − 2.52, − 0.32, p value: 0.012) and regret of choice of nursing studies (RC: 2.13, 95% CI 0.58, 3.68, p value = 0.007) were found to be independent predictors of burnout in these students. Early identification of these determinants is required to prevent progression to burnout.

**Electronic supplementary material:**

The online version of this article (10.1186/s13104-018-3567-3) contains supplementary material, which is available to authorized users.

## Introduction

Burnout syndrome is a psychological state characterised by emotional exhaustion and distancing one’s self from their job (cynicism) [1]. It usually results from job stressors and is highly prevalent amongst medical personnel [2, 3]. It is widely accepted that due to the strenuous academic life of medical students characterised by competitive entrance exams, difficult transitions across the various phases of clinical work and the exposure to critically ill patients; these students are quite likely to experience burnout syndrome [4, 5]. In Cameroon, students who wish to obtain a nursing degree have similar exposures as enumerated above. There are only two state-owned nursing schools in the English-speaking regions in Cameroon, which provide nursing degrees with highly competitive entrance exams. It is also widely accepted that burnout syndrome starts at the early stages of training and worsens during practice with work environment stressors; and increased workload being a huge contributing factor [6, 7].

Researchers have found a relatively high prevalence of burnout amongst nursing students. In Brazil, a study showed that 64.04% of nursing students in the sample had emotional exhaustion, 35.79% had a high level of cynicism, and 87.72% had a low level of professional efficacy [8]. There is an acute shortage of nurses in Cameroon with a nurses’ density of 0.67 per 1000 [9]. With such a workload awaiting nursing students with burnout, it is necessary for early detection of the syndrome as burnout in known to reduce optimal healthcare delivery. If the determinants of burnout can be identified amongst these students and early detection made a priority, the morbidity associated with this condition could be reduced.

This research aims to raise awareness on mental health issues amongst these group of students in a field where there is usually a dearth of data. This could help inform public health policy to help with the investigation and institution of adequate preventive measures.

## Main text

### Methods

#### Population and design

##### Population, sampling and sample size

This manuscript was written from data obtained from a wider study assessing burnout and depression in nursing students in Cameroon (the Me-HeLTC project). The sampling frame included all the nursing students from the two state-owned nursing schools (University of Bamenda and University of Buea) and other private nursing schools (Saint Veronica higher institute of biomedical and nursing sciences Buea and Saint Louis higher institute of health Bamenda); in the two English-speaking regions of the country. These students were recruited by a convenience sampling until the minimum sample size required was attained which was calculated by the formula below [10]:

$$n = \frac{{{Z^2}\;P\;\left( {1 - P} \right)}}{d^2}$$

where n = sample size, Z = Z statistic for a level of confidence, P = expected prevalence or proportion (in proportion of one: 27.4, P = 0.274) [11], and d = precision (in proportion of one; if 5%, d = 0.05). Z statistic (Z): For the level of confidence of 95%, which is conventional, Z value is 1.96. For a 95% confidence intervals (CI).

A minimum of 306 nursing students were required for the study and 447 questionnaires were handed out and returned filled.

##### Design

This was a cross-sectional study that was carried out over 4 months (January to April 2018).

##### Instrument

The following information was collected:

Socio-demographic characteristics: age; sex; marital status; number of children; level of nursing studies; number of hours spent studying; presence of problems in personal relationships (defined as a close connection between two people formed by emotional and sexual interactions); monthly income and the perception of sustainability on their monthly income; average academic results attained; alcohol use; (assessed using units of alcohol consumed a week); smoking and history of any chronic disease (which included asthma, chronic pelvic pain, hypertension, diabetes mellitus, gastroesophageal reflux disease, chronic peptic ulcer disease, migraines, cancers, HIV/AIDS, cerebral lesions, chronic liver and kidney disease. An open-ended question was also provided for the students to state their chronic illness which was used by the investigators to assess the suitability of the response for this scenario).

The OLdenburg Burnout Inventory (OLBI) which is a self-administered questionnaire consisting of 16 positively and negatively framed questions to assess two core parameters of burnout with eight questions for each parameter: exhaustion (intense physical, affective and cognitive strain) and disengagement (characterized as cynicism referring to distancing from one’s work in general) was used to assess burnout syndrome [1]. The OLBI version used in this study was adapted from that used by Reis et al. [12] in an academic milieu. The OLBI ranges on a scale of one to four from strongly agree to strongly disagree.

#### Data management and statistical analysis

Data entry was done using EPI Info version 7 (CDC, Atlanta). Accuracy of data entry was assessed by crosschecking a random sample of 10% of the data.

Results were presented as proportions and means with standard deviations (SD). The reliability and validity of the OLBI was assessed by means of Cronbach alpha coefficients.

Emotional exhaustion and disengagement as defined by the OLBI were used to define burnout. Negatively phrased questions were reversed (1 = 4, 2 = 3, 3 = 2, 4 = 1) such that higher scores indicated higher exhaustion and disengagement and the values entered by each participant for both dimensions were summed up to obtain a total score for burnout syndrome.

Univariable linear regression analysis was used to compare the outcome (burnout syndrome) with potential determinants such as: marital status; sex; age; level of studies; presence of a chronic illness; perceived poor academic performance; alcohol consumption and smoking. Significant predictors were inputted in a multivariable linear regression model to identify independent determinants of burnout syndrome. Significance was set at p values < 0.05.

### Results

#### General characteristics

A total of 447 questionnaires were distributed and returned filled from the nursing students. There were 99 (22.45%) students from the University of Bamenda, 91 (20.63%) from the University of Buea and 251 (56.92%) from the private nursing schools (Saint Veronica higher institute of biomedical and nursing sciences Buea and Saint Louis higher institute of health Bamenda). Most (81.17%) of the students were female with a majority (39.73%) being in their second year of studies (Table 1). The ages of the students ranged from 17 to 50 years with a mean age of 22.28 ± 3.61 (Table 2).

**Table 1:** Categorical variables showing the sociodemographic characteristics of 447 nursing students in the English-speaking regions of Cameroon assessed for depression from January to April 2018

Variable	Total	
	N	%
Nursing school (n = 441)		
University of Bamenda	99	22.45
University of Buea	91	20.63
Private sector	251	56.92
Level of studies (n = 443)		
Year 1	123	27.77
Year 2	176	39.73
Year 3	91	20.54
Year 4	53	11.96
Gender (n = 446)		
Male	84	18.83
Female	362	81.17
Personal relationship (n = 440)^a^		
Yes	221	50.23
No	219	49.77
Marital status (n = 446)		
Single	401	89.91
Married	45	10.09
Difficulties in personal relationship (n = 427)		
Yes	77	18.03
No	350	81.97
Exams re-sited (n = 429)		
Yes	220	51.28
No	209	48.72
Courses repeated (n = 439)		
Yes	168	38.27
No	271	61.73
Satisfaction with results (n = 396)		
Yes	122	30.81
No	274	69.19
Regret choice of nursing studies (n = 439)		
Yes	50	11.39
No	389	88.61
Occurrence of life changing crises in last 6 months (n = 434)^b^		
Yes	221	50.92
No	213	49.08
Presence of chronic illness (n = 445)^c^		
Yes	19	4.27
No	426	95.73
Alcohol consumption (n = 446)		
Yes	127	28.48
No	319	71.52
Recreational drug use (n = 444)^d^		
Yes	5	1.13
No	439	98.87
Sufficient monthly income (n = 407)		
Yes	98	24.08
No	309	75.92

^a^ Personal relationship was defined as close connections between two people formed by emotional and sexual interactions

^b^ Life changing crises defined as loss of a loved one, physical or sexual trauma and conditions of emotional or social instability

^c^ Chronic illnesses included: Asthma, chronic pelvic pain, diabetes mellitus, gastroesophageal reflux disease, chronic peptic ulcer disease, migraines, cerebral lesions and paralysis

^d^ Recreational drugs included: marijuana and tramadol

**Table 2:** Continuous variables showing the sociodemographic characteristics of 447 nursing students in the English-speaking regions of Cameroon assessed for depression from January to April 2018

Variable	Number of observations	Total sample			
		Mean	SD	Min	Max
Age	400	22.28	3.61	17	50
Number of hours studying	425	4.74	3.10	0	19
Monthly income in USD	287	32.85	23.46	0	187.68
GPA	306	2.79	0.59	0	4

*USD* United states dollars, *GPA* cumulative grade point average, *OLBI* Oldenburg burnout inventory

#### Assessment of burnout syndrome

The average for disengagement items was 17.10 ± 3.09 (minimum = 8, maximum = 26) while the average for exhaustion items was 20.94 ± 3.04 (minimum = 13, maximum = 31). All the items showed average covariances and the alpha Cronbach coefficient was 0.6 showing that the OLBI measured the same underlying construct of burnout amongst the participants (Additional file 1).

#### Determinants of burnout syndrome

On univariable linear regression analysis, burnout syndrome was associated with: marital status [regression coefficient (RC): 6.19; 95% confidence intervals (CI) 2.82, 9.56, p value: < 0.001]; relationship difficulties (RC: 2.55, 95% CI 1.05, 4.05; p value: 0.001); number of children (RC: − 0.89; 95% CI − 1.55, − 0.23; p value: 0.008); level of studies (RC: 2.05, 95% CI 0.91, 3.20; p value: < 0.001); satisfaction with results (RC: − 1.17; 95% CI − 2.21, − 0.13; p value: 0.028); and regret of choice of nursing studies (RC: 2.30; 95% CI 0.91, 3.69; p value: 0.001) (Table 3).

**Table 3:** Univariable linear regression analysis of potential determinants of burnout syndrome amongst 447 nursing students who were assessed for burnout syndrome from January to April 2018 in the two English-speaking regions of Cameroon

Variables	Coefficient	Intercept	95% CI	p value
Age	− 0.16	41.71	− 0.30, − 0.04	0.012
Gender (female/male)	0.21	37.87	− 0.94, 1.35	0.723
Marital status (married/single)	6.19	36.98	2.82, 9.56	< 0.001
Difficulties in personal relationships (yes/no)	1.09	37.81	− 0.09, 2.27	0.069
Number of children	− 0.89	38.20	− 1.55, − 0.23	0.008
Level of studies	− 0.07	38.15	− 0.54, 0.39	0.760
Number of hours studying	− 0.09	38.40	− 0.24, 0.06	0.241
Monthly income (in USD)	0.02	37.52	− 0.01, 0.04	0.135
Sufficient monthly income (yes/no)	− 0.63	38.29	− 1.74, 0.48	0.266
Cumulative GPA	− 0.54	39.50	− 1.48, 0.41	0.265
Satisfaction with results (yes/no)	− 1.17	38.45	− 2.21, − 0.13	0.028
Regret choice of nursing studies (yes/no)	2.30	37.74	0.91, 3.69	0.001
Recreational drug use (yes/no)	3.59	38.01	− 0.63, 7.81	0.096
Presence of chronic illness (yes/no)	1.59	37.99	− 0.61, 3.79	0.157
Life changing crises (yes/no)	0.60	37.80	− 0.31, 1.50	0.195

After multivariable linear regression analysis, the following variables were identified to be independent determinants of burnout: satisfaction with results (RC: − 1.42, 95% CI − 2.52, − 0.32, p value: 0.012) and regret of choice of nursing studies (RC: 2.13, 95% CI 0.58, 3.68, p value = 0.007) (Additional file 2).

### Discussion

In this study, we showed that independent determinants of burnout syndrome amongst nursing students in the two English-speaking regions of Cameroon were dissatisfaction with results and regret of choice of nursing studies.

Students who regretted choosing nursing as a future career scored on average two points higher on the OLBI scale after adjusting for confounders. Burnout syndrome has been associated with the intention of giving up nursing studies among nursing students in the South of Brazil [13] and amongst students in other fields like medicine elsewhere [5, 14, 15]. Nursing studies in Cameroon are particularly stressful. The entrance examinations into the two state universities are very competitive and the private sector which offers a few more opportunities are quite expensive to pay for. The studies are also time consuming, with students having to sit through several hours of classes per week; which happens to be exhausting both mentally and physically. As such, they end up having little or no time to engage in social and pleasurable activities which they enjoy; and which have been shown to have not only mental but equally physical benefits [16, 17]. This could lead to regrets and ultimately burnout as pointed out by our analysis.

Furthermore, students who get into these nursing programmes are faced with the harsh realities of autonomous university life, the difficulties of transitioning in the basic nursing sciences and the daily dilemmas of dealing with critically-ill patients during their routine clinical clerkships. They also begin to spend less time at home with family as they begin to run shifts during their internships; are faced with big emotional tests as they start to lose patients under their care; and equally get to realize the workload associated with nursing in Cameroon due to the acute shortage of nurses in the country (nurses’ density: 0.67 per 1000) [9]. With all the above mentioned difficulties linked to nursing studies in Cameroon, it is understandable that some of these students may start to feel uncomfortable with their choice of studies especially if they have the feeling of not coping in this environment and lack the hardiness to survive the coursework. Such feelings could easily perpetuate further an already toxic academic milieu [15] which could easily lead to them becoming burnt out. Both environmental and personal factors can lead to stress among students, previous studies have shown an association between certain personality traits such as resilience and burnout syndrome in nursing students [8, 11].

A vast range of career opportunities usually awaits high school leavers but the nursing field is a popular choice in Cameroon due to its high employability and apparent financial safety. The realities of the nursing studies and a career in nursing are hardly made available to prospective students. Students who are ill-equipped to deal with such pressure may end up making a poor career choice. Proper orientation is therefore necessary for these students to make career choices that match their skill set and expectancies.

The above situation may become more relevant if the performance of the student becomes affected by this environment. Studies have shown that dissatisfaction with results is significantly associated with stress [18] and depression [19] among students in the health training system. This has a negative impact on students as stress or burnout may result in a decrease in performance of daily educational tasks and poor results could ensue. This leads to dissatisfaction with performance and results, and more stress about their abilities to succeed in the chosen field. This vicious cycle continues throughout their studies which could eventually lead to burnout. Indeed, in this study, students who were dissatisfied with their results significantly scored at least one point more on the OLBI scale than students who were satisfied with their results. As earlier mentioned, nursing studies in Cameroon are not only stressful but also time consuming. As a result, students have little or no opportunity to accomplish leisure time activities. The inability to accomplish such activities because of the time consuming nature of their studies could therefore weigh on their mental health status.

We did not find a significant association between burnout syndrome and alcohol consumption, recreational drug use and tobacco use respectively. These findings are similar to a study among nurses in Brazil, although high levels of burnout and risk turnover may lead to alcohol or recreational drug use as a means of coping with stress [20]. However, this study was carried out in a conservative society where recreational drug use is highly frowned upon. Students may not have indicated previous drug use to appear more socially desirable. Also, over half of the students had experienced a significant life changing crises in the past 6 months. Though this was not associated with burnout syndrome in these students, it highlights the additional strain nursing students undergo alongside their studies.

Though mental health is usually neglected in most low and middle-income countries with health professionals being ill-equipped to deal with mental health conditions especially in Cameroon [21], burnout is gaining global recognition as a psychological state that affects a higher proportion of medical personnel and trainees compared to the general public [22–25]. There is great need for provision of counselling and support services in nursing programs and the utilization of effective interventions and coping strategies to prevent or reduce burnout syndrome and augment academic motivation. With the dearth of data on this issue in Cameroon this study provides determinants of this condition amongst a special population that may help raise awareness on the need for early identification of this determinants and management to prevent progression to burnout syndrome. This could help decrease the associated morbidity and inform policymakers on the need for the investigation and institution of appropriate preventive measures.

## Limitations

This is the first study to the best of our knowledge which assesses the determinants of burnout syndrome amongst nursing students in Cameroon and in Central Africa. However, due to the cross-sectional design of the study and the use of self-reported questionnaires the study was subjected to recall bias.

## Additional files

**Additional file 1.** Covariances of various items on the OLBI and the associated alpha Cronbach coefficient of 0.6 showing that the items measure the same underlying construct—burnout.

**Additional file 2.** Multivariable linear regression analysis of potential determinants of burnout syndrome amongst 413 medical students who were assessed for burnout syndrome from January–March 2018 in Cameroon.

## Abbreviations

OLBI: OLdenburg Burnout Inventory
GPA: grade point average
SE: standard error
SD: standard deviation
CI: confidence interval
