# Supplementary material for: Determinants of burnout syndrome among nursing students in Cameroon: cross-sectional study
Source: BMC Res Notes. 2018 Jul 9;11:450. doi: 10.1186/s13104-018-3567-3 (PMC6038299; doi:10.1186/s13104-018-3567-3)
Supplement: Supplementary file 1 — Additional file 1. Covariances of various items on the OLBI and the associated alpha Cronbach coefficient of 0.6 showing that the items measure the same underlying construct—burnout. [file 13104_2018_3567_MOESM1_ESM.docx]

| **Item** | **Average inter-item covariance** | **Alpha Cronbach coefficient** |
| --- | --- | --- |
| I always find new and interesting aspects in my studies (D) | 0.06 | 0.6 |
| It happens more and more often that I talk about my studies in a negative way (D) (R) | 0.05 | 0.6 |
| Lately, I tend to think less about my academic tasks and do them almost mechanically (D) | 0.05 | 0.6 |
| I find my studies to be a positive challenge (D) | 0.06 | 0.6 |
| Over time, one can become disconnected from this type of studies (D) (R) | 0.05 | 0.6 |
| Sometimes I feel sickened by my studies (D) (R) | 0.05 | 0.6 |
| Medicine is the only field of study that I can imagine myself doing (D) | 0.06 | 0.6 |
| I feel more and more engaged in my studies (D) | 0.05 | 0.6 |
| There are days when I feel tired before I arrive in class or start studying (E) (R) | 0.06 | 0.6 |
| After a class or after studying, I tend to need more time than in the past in order to relax and feel better (E) (R) | 0.06 | 0.6 |
| I can tolerate the pressure of my studies very well (E) | 0.05 | 0.6 |
| While studying, I often feel emotionally drained (E) (R) | 0.06 | 0.6 |
| After a class or after studying, I have enough energy for my leisure activities (E) | 0.06 | 0.6 |
| After a class or after studying, I usually feel worn out and weary (E) (R) | 0.06 | 0.6 |
| I can usually manage my study-related workload well (E) | 0.06 | 0.6 |
| When I study, I usually feel energized (E) | 0.06 | 0.6 |
| Test scale |  | 0.6 |

D: Disengagement items; R: Items that were reversed before analyses; E: Exhaustion items
