# Supplementary material for: Determinants of burnout syndrome among nursing students in Cameroon: cross-sectional study
Source: BMC Res Notes. 2018 Jul 9;11:450. doi: 10.1186/s13104-018-3567-3 (PMC6038299; doi:10.1186/s13104-018-3567-3)
Supplement: Supplementary file 2 — Additional file 2. Multivariable linear regression analysis of potential determinants of burnout syndrome amongst 413 medical students who were assessed for burnout syndrome from January–March 2018 in Cameroon. [file 13104_2018_3567_MOESM2_ESM.docx]

| **Determinants** | **Coefficient** | **95% Confidence interval** | **p value** |
| --- | --- | --- | --- |
| Age | -0.09 | -0.27, 0.08 | 0.291 |
| Marital status (Married/Single) | -0.57 | -2.56, 1.41 | 0.569 |
| Number of children | -0.35 | -1.33, 0.64 | 0.488 |
| Satisfaction with results (Yes/No) | -1.42 | -2.52, -0.32 | 0.012 |
| Regret choice of nursing studies (Yes/No) | 2.13 | 0.58, 3.68 | 0.007 |
| Intercept | 40.42 | 36.57, 44.27 |  |
